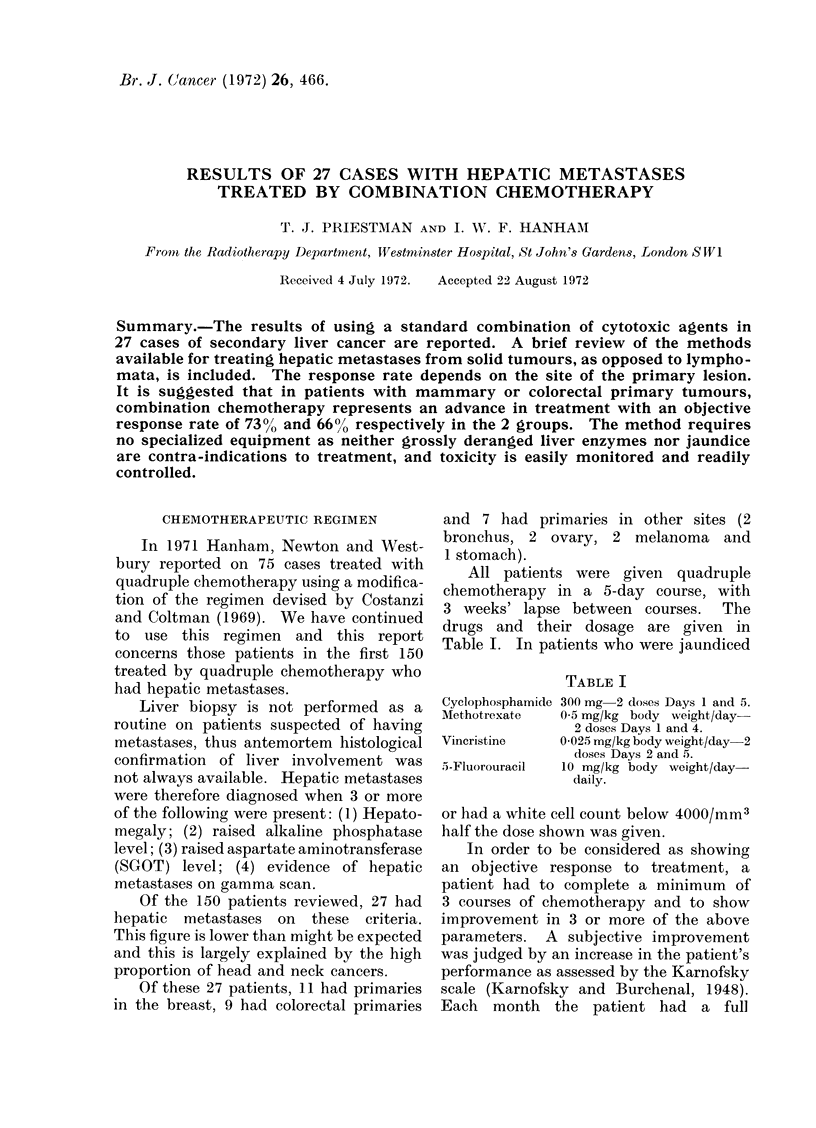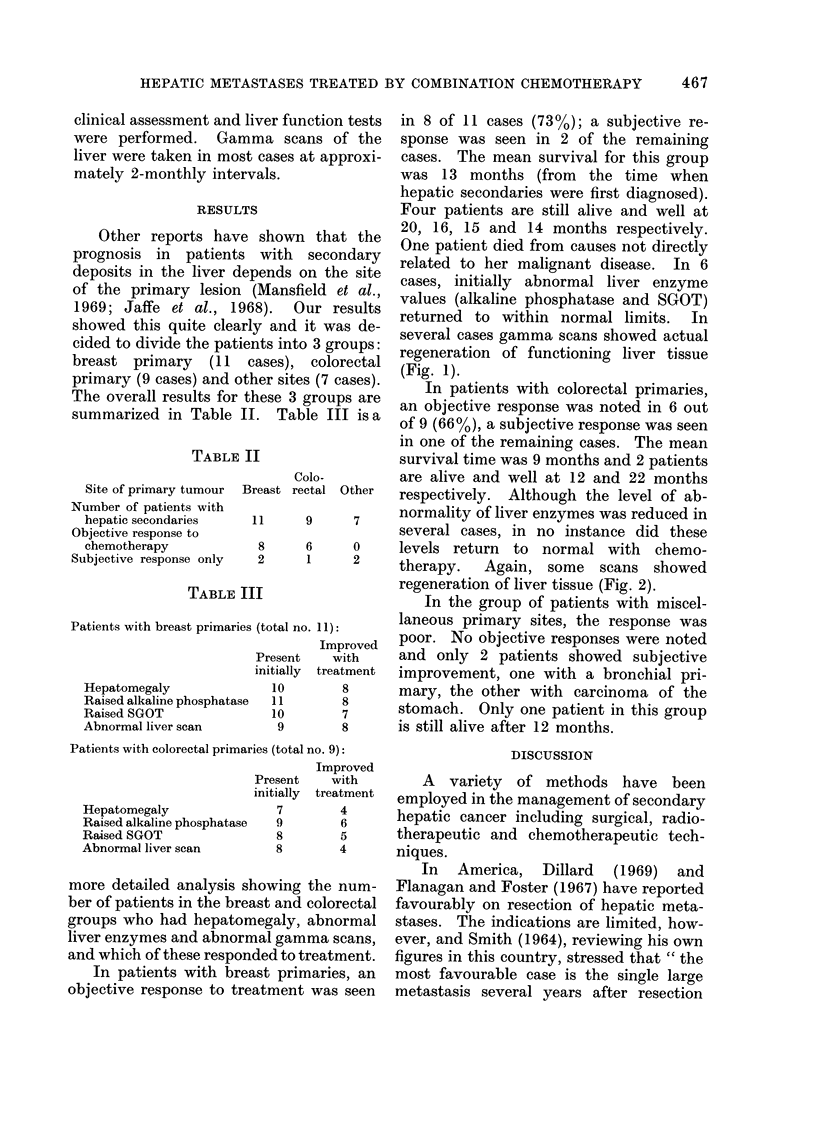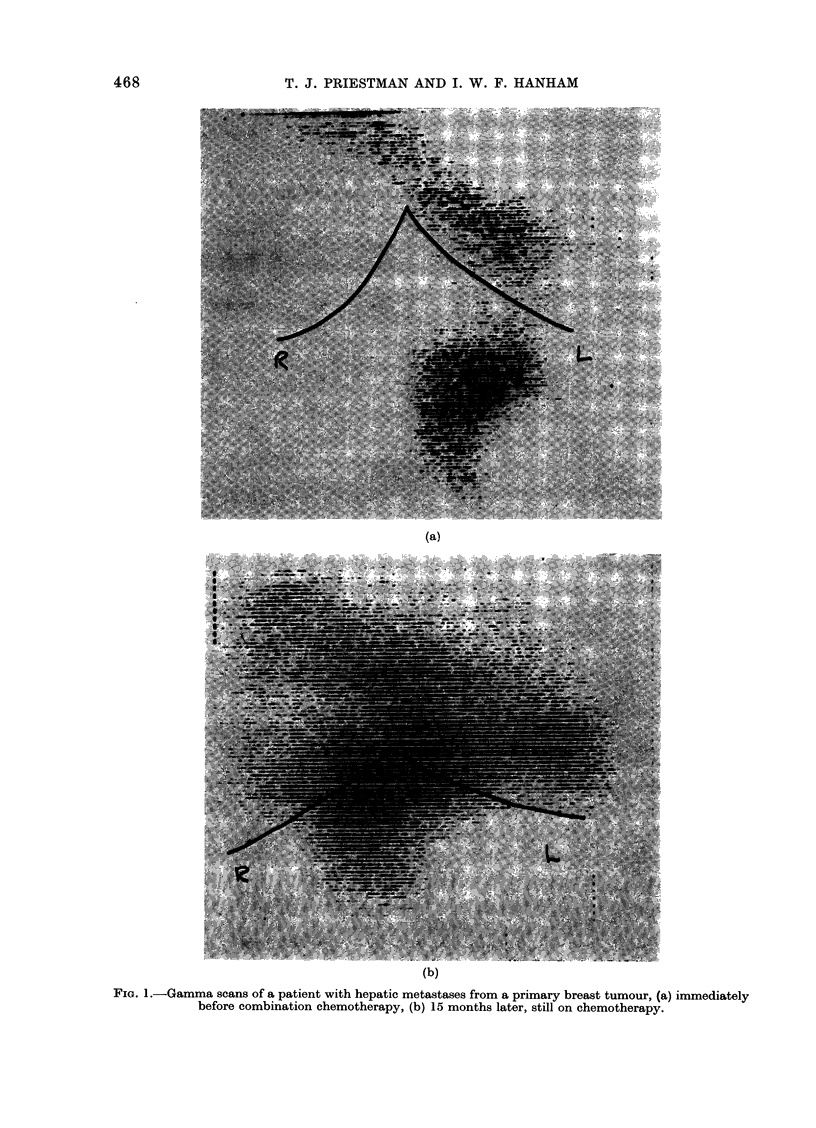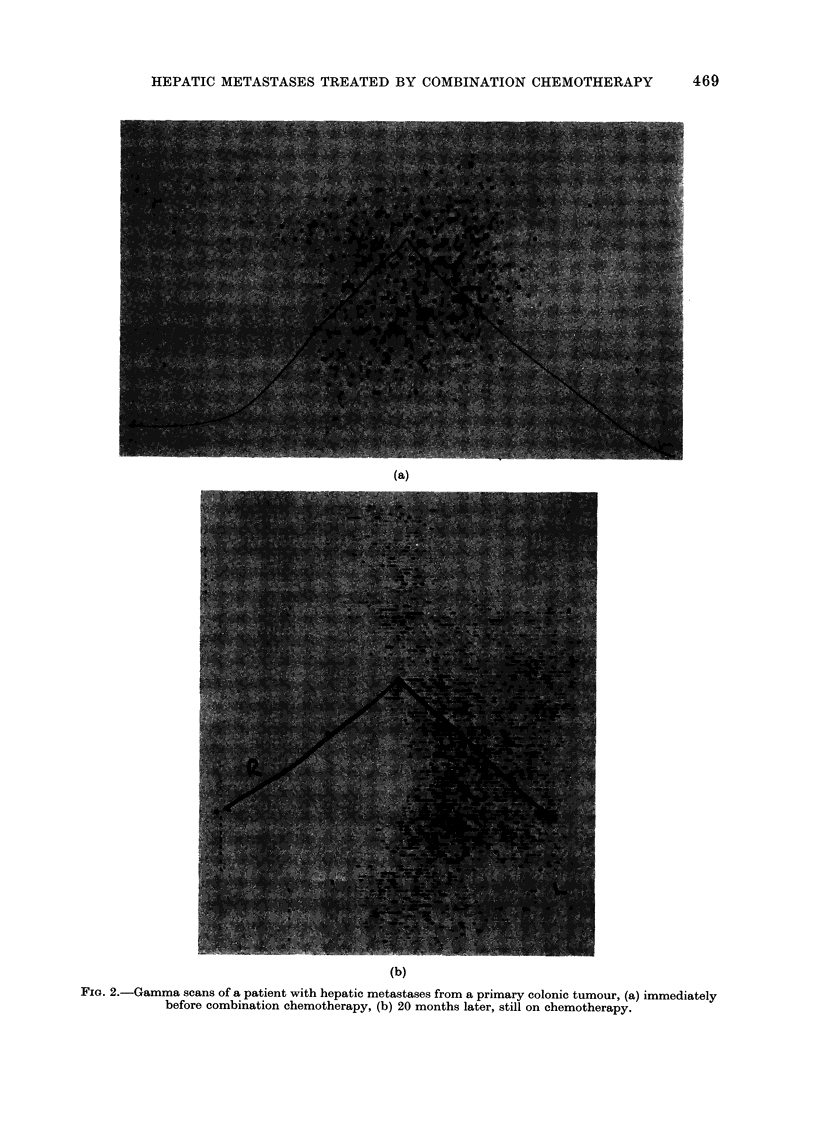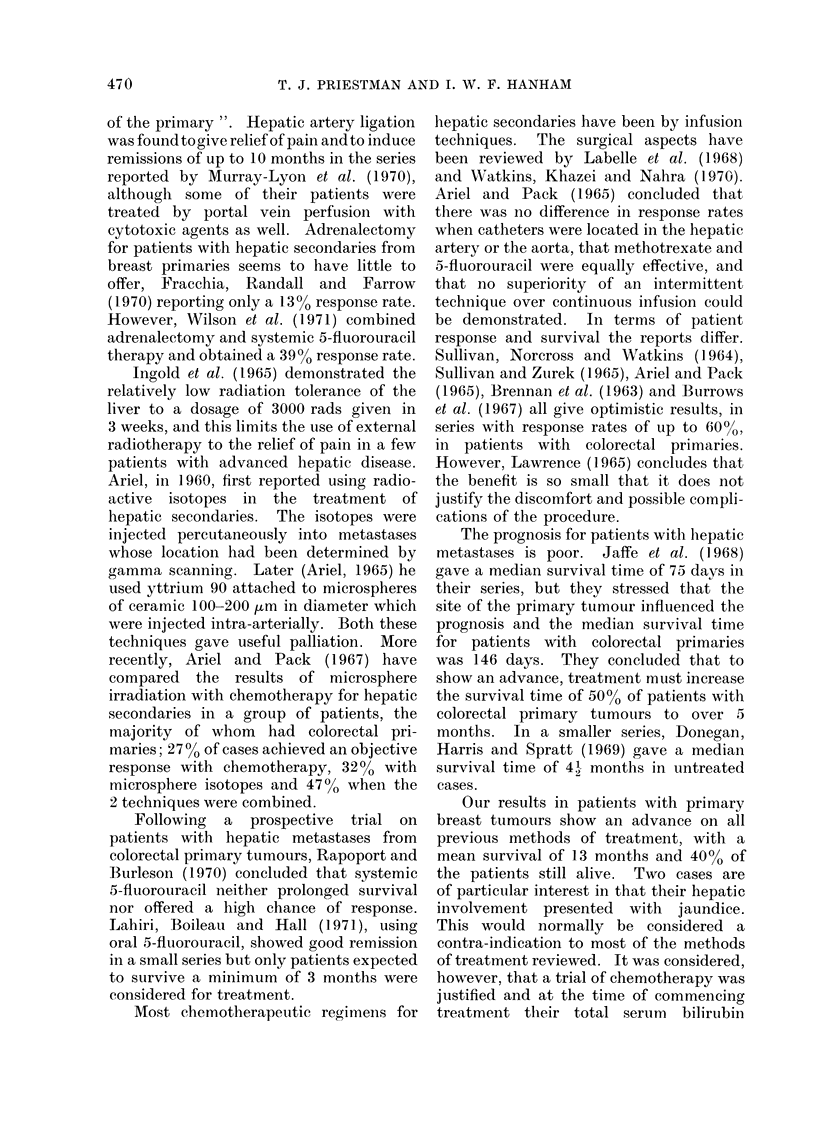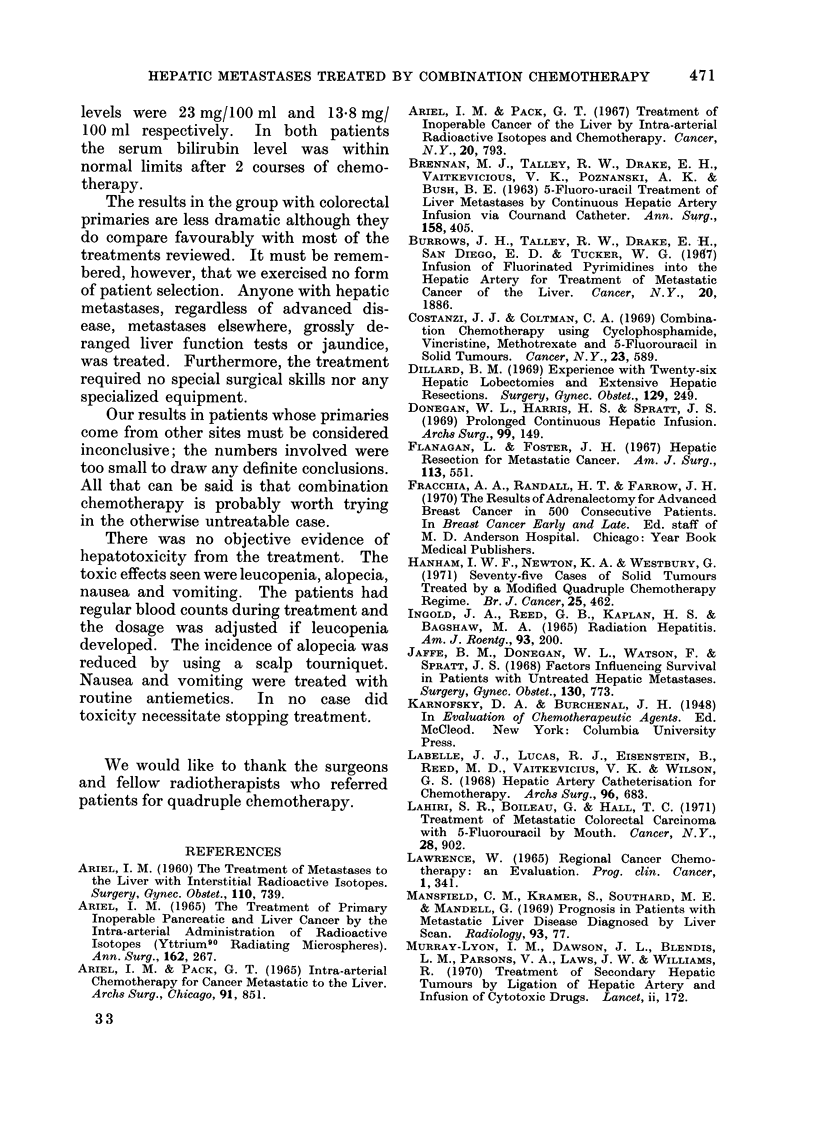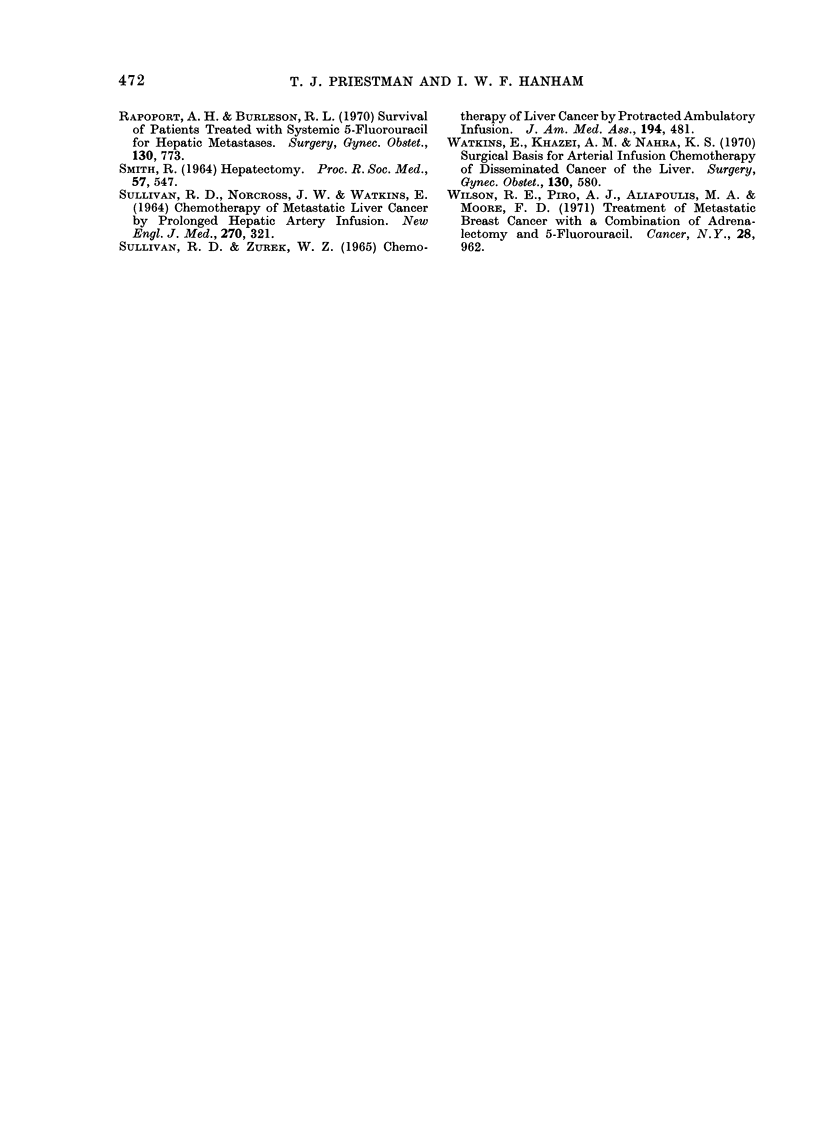# Results of 27 Cases with Hepatic Metastases Treated by Combination Chemotherapy

**DOI:** 10.1038/bjc.1972.64

**Published:** 1972-12

**Authors:** T. J. Priestman, I. W. F. Hanham

## Abstract

**Images:**


*Br. J. Cancer* (1972) **26**, 466.

### RESULTS OF 27 CASES WITH HEPATIC METASTASES TREATED BY COMBINATION CHEMOTHERAPY

T. J. PRIESTMAN AND I. W. F. HANHAM

*From the Radiotherapy Department, Westminster Hospital, St John's Gardens, London SW1*

Received 4 July 1972. Accepted 22 August 1972

**Summary.—The results of using a standard combination of cytotoxic agents in 27 cases of secondary liver cancer are reported. A brief review of the methods available for treating hepatic metastases from solid tumours, as opposed to lymphomata, is included. The response rate depends on the site of the primary lesion. It is suggested that in patients with mammary or colorectal primary tumours, combination chemotherapy represents an advance in treatment with an objective response rate of 73% and 66% respectively in the 2 groups. The method requires no specialized equipment as neither grossly deranged liver enzymes nor jaundice are contra-indications to treatment, and toxicity is easily monitored and readily controlled.**

### CHEMOTHERAPEUTIC REGIMEN

In 1971 Hanham, Newton and Westbury reported on 75 cases treated with quadruple chemotherapy using a modification of the regimen devised by Costanzi and Coltman (1969). We have continued to use this regimen and this report concerns those patients in the first 150 treated by quadruple chemotherapy who had hepatic metastases.

Liver biopsy is not performed as a routine on patients suspected of having metastases, thus antemortem histological confirmation of liver involvement was not always available. Hepatic metastases were therefore diagnosed when 3 or more of the following were present: (1) Hepatomegaly; (2) raised alkaline phosphatase level; (3) raised aspartate aminotransferase (SGOT) level; (4) evidence of hepatic metastases on gamma scan.

Of the 150 patients reviewed, 27 had hepatic metastases on these criteria. This figure is lower than might be expected and this is largely explained by the high proportion of head and neck cancers.

Of these 27 patients, 11 had primaries in the breast, 9 had colorectal primaries and 7 had primaries in other sites (2 bronchus, 2 ovary, 2 melanoma and 1 stomach).

All patients were given quadruple chemotherapy in a 5-day course, with 3 weeks' lapse between courses. The drugs and their dosage are given in Table I. In patients who were jaundiced or had a white cell count below 4000/mm$^3$ half the dose shown was given.

TABLE I

| | |
|---|---|
| Cyclophosphamide | 300 mg—2 doses Days 1 and 5. |
| Methotrexate | 0·5 mg/kg body weight/day—2 doses Days 1 and 4. |
| Vincristine | 0·025 mg/kg body weight/day—2 doses Days 2 and 5. |
| 5-Fluorouracil | 10 mg/kg body weight/day—daily. |

In order to be considered as showing an objective response to treatment, a patient had to complete a minimum of 3 courses of chemotherapy and to show improvement in 3 or more of the above parameters. A subjective improvement was judged by an increase in the patient's performance as assessed by the Karnofsky scale (Karnofsky and Burchenal, 1948). Each month the patient had a full clinical assessment and liver function tests were performed. Gamma scans of the liver were taken in most cases at approximately 2-monthly intervals.

### RESULTS

Other reports have shown that the prognosis in patients with secondary deposits in the liver depends on the site of the primary lesion (Mansfield *et al.*, 1969; Jaffe *et al.*, 1968). Our results showed this quite clearly and it was decided to divide the patients into 3 groups: breast primary (11 cases), colorectal primary (9 cases) and other sites (7 cases). The overall results for these 3 groups are summarized in Table II. Table III is a more detailed analysis showing the number of patients in the breast and colorectal groups who had hepatomegaly, abnormal liver enzymes and abnormal gamma scans, and which of these responded to treatment.

TABLE II

| Site of primary tumour | Breast | Colo-rectal | Other |
|---|---|---|---|
| Number of patients with hepatic secondaries | 11 | 9 | 7 |
| Objective response to chemotherapy | 8 | 6 | 0 |
| Subjective response only | 2 | 1 | 2 |

TABLE III

Patients with breast primaries (total no. 11):

| | Present initially | Improved with treatment |
|---|---|---|
| Hepatomegaly | 10 | 8 |
| Raised alkaline phosphatase | 11 | 8 |
| Raised SGOT | 10 | 7 |
| Abnormal liver scan | 9 | 8 |

Patients with colorectal primaries (total no. 9):

| | Present initially | Improved with treatment |
|---|---|---|
| Hepatomegaly | 7 | 4 |
| Raised alkaline phosphatase | 9 | 6 |
| Raised SGOT | 8 | 5 |
| Abnormal liver scan | 8 | 4 |

In patients with breast primaries, an objective response to treatment was seen in 8 of 11 cases (73%); a subjective response was seen in 2 of the remaining cases. The mean survival for this group was 13 months (from the time when hepatic secondaries were first diagnosed). Four patients are still alive and well at 20, 16, 15 and 14 months respectively. One patient died from causes not directly related to her malignant disease. In 6 cases, initially abnormal liver enzyme values (alkaline phosphatase and SGOT) returned to within normal limits. In several cases gamma scans showed actual regeneration of functioning liver tissue (Fig. 1).

In patients with colorectal primaries, an objective response was noted in 6 out of 9 (66%), a subjective response was seen in one of the remaining cases. The mean survival time was 9 months and 2 patients are alive and well at 12 and 22 months respectively. Although the level of abnormality of liver enzymes was reduced in several cases, in no instance did these levels return to normal with chemotherapy. Again, some scans showed regeneration of liver tissue (Fig. 2).

In the group of patients with miscellaneous primary sites, the response was poor. No objective responses were noted and only 2 patients showed subjective improvement, one with a bronchial primary, the other with carcinoma of the stomach. Only one patient in this group is still alive after 12 months.

### DISCUSSION

A variety of methods have been employed in the management of secondary hepatic cancer including surgical, radiotherapeutic and chemotherapeutic techniques.

In America, Dillard (1969) and Flanagan and Foster (1967) have reported favourably on resection of hepatic metastases. The indications are limited, however, and Smith (1964), reviewing his own figures in this country, stressed that " the most favourable case is the single large metastasis several years after resection

Fig. 1.—Gamma scans of a patient with hepatic metastases from a primary breast tumour, (a) immediately before combination chemotherapy, (b) 15 months later, still on chemotherapy.

FIG. 2.—Gamma scans of a patient with hepatic metastases from a primary colonic tumour, (a) immediately before combination chemotherapy, (b) 20 months later, still on chemotherapy.

of the primary ". Hepatic artery ligation was found to give relief of pain and to induce remissions of up to 10 months in the series reported by Murray-Lyon *et al.* (1970), although some of their patients were treated by portal vein perfusion with cytotoxic agents as well. Adrenalectomy for patients with hepatic secondaries from breast primaries seems to have little to offer, Fracchia, Randall and Farrow (1970) reporting only a 13% response rate. However, Wilson *et al.* (1971) combined adrenalectomy and systemic 5-fluorouracil therapy and obtained a 39% response rate.

Ingold *et al.* (1965) demonstrated the relatively low radiation tolerance of the liver to a dosage of 3000 rads given in 3 weeks, and this limits the use of external radiotherapy to the relief of pain in a few patients with advanced hepatic disease. Ariel, in 1960, first reported using radioactive isotopes in the treatment of hepatic secondaries. The isotopes were injected percutaneously into metastases whose location had been determined by gamma scanning. Later (Ariel, 1965) he used yttrium 90 attached to microspheres of ceramic 100–200 $\mu$m in diameter which were injected intra-arterially. Both these techniques gave useful palliation. More recently, Ariel and Pack (1967) have compared the results of microsphere irradiation with chemotherapy for hepatic secondaries in a group of patients, the majority of whom had colorectal primaries; 27% of cases achieved an objective response with chemotherapy, 32% with microsphere isotopes and 47% when the 2 techniques were combined.

Following a prospective trial on patients with hepatic metastases from colorectal primary tumours, Rapoport and Burleson (1970) concluded that systemic 5-fluorouracil neither prolonged survival nor offered a high chance of response. Lahiri, Boileau and Hall (1971), using oral 5-fluorouracil, showed good remission in a small series but only patients expected to survive a minimum of 3 months were considered for treatment.

Most chemotherapeutic regimens for hepatic secondaries have been by infusion techniques. The surgical aspects have been reviewed by Labelle *et al.* (1968) and Watkins, Khazei and Nahra (1970). Ariel and Pack (1965) concluded that there was no difference in response rates when catheters were located in the hepatic artery or the aorta, that methotrexate and 5-fluorouracil were equally effective, and that no superiority of an intermittent technique over continuous infusion could be demonstrated. In terms of patient response and survival the reports differ. Sullivan, Norcross and Watkins (1964), Sullivan and Zurek (1965), Ariel and Pack (1965), Brennan *et al.* (1963) and Burrows *et al.* (1967) all give optimistic results, in series with response rates of up to 60%, in patients with colorectal primaries. However, Lawrence (1965) concludes that the benefit is so small that it does not justify the discomfort and possible complications of the procedure.

The prognosis for patients with hepatic metastases is poor. Jaffe *et al.* (1968) gave a median survival time of 75 days in their series, but they stressed that the site of the primary tumour influenced the prognosis and the median survival time for patients with colorectal primaries was 146 days. They concluded that to show an advance, treatment must increase the survival time of 50% of patients with colorectal primary tumours to over 5 months. In a smaller series, Donegan, Harris and Spratt (1969) gave a median survival time of $4\frac{1}{2}$ months in untreated cases.

Our results in patients with primary breast tumours show an advance on all previous methods of treatment, with a mean survival of 13 months and 40% of the patients still alive. Two cases are of particular interest in that their hepatic involvement presented with jaundice. This would normally be considered a contra-indication to most of the methods of treatment reviewed. It was considered, however, that a trial of chemotherapy was justified and at the time of commencing treatment their total serum bilirubin levels were 23 mg/100 ml and 13·8 mg/100 ml respectively. In both patients the serum bilirubin level was within normal limits after 2 courses of chemotherapy.

The results in the group with colorectal primaries are less dramatic although they do compare favourably with most of the treatments reviewed. It must be remembered, however, that we exercised no form of patient selection. Anyone with hepatic metastases, regardless of advanced disease, metastases elsewhere, grossly deranged liver function tests or jaundice, was treated. Furthermore, the treatment required no special surgical skills nor any specialized equipment.

Our results in patients whose primaries come from other sites must be considered inconclusive; the numbers involved were too small to draw any definite conclusions. All that can be said is that combination chemotherapy is probably worth trying in the otherwise untreatable case.

There was no objective evidence of hepatotoxicity from the treatment. The toxic effects seen were leucopenia, alopecia, nausea and vomiting. The patients had regular blood counts during treatment and the dosage was adjusted if leucopenia developed. The incidence of alopecia was reduced by using a scalp tourniquet. Nausea and vomiting were treated with routine antiemetics. In no case did toxicity necessitate stopping treatment.

We would like to thank the surgeons and fellow radiotherapists who referred patients for quadruple chemotherapy.

### REFERENCES

ARIEL, I. M. (1960) The Treatment of Metastases to the Liver with Interstitial Radioactive Isotopes. *Surgery, Gynec. Obstet.*, **110**, 739.

ARIEL, I. M. (1965) The Treatment of Primary Inoperable Pancreatic and Liver Cancer by the Intra-arterial Administration of Radioactive Isotopes (Yttrium$^{90}$ Radiating Microspheres). *Ann. Surg.*, **162**, 267.

ARIEL, I. M. & PACK, G. T. (1965) Intra-arterial Chemotherapy for Cancer Metastatic to the Liver. *Archs Surg., Chicago*, **91**, 851.

ARIEL, I. M. & PACK, G. T. (1967) Treatment of Inoperable Cancer of the Liver by Intra-arterial Radioactive Isotopes and Chemotherapy. *Cancer, N.Y.*, **20**, 793.

BRENNAN, M. J., TALLEY, R. W., DRAKE, E. H., VAITKEVICIOUS, V. K., POZNANSKI, A. K. & BUSH, B. E. (1963) 5-Fluoro-uracil Treatment of Liver Metastases by Continuous Hepatic Artery Infusion via Cournand Catheter. *Ann. Surg.*, **158**, 405.

BURROWS, J. H., TALLEY, R. W., DRAKE, E. H., SAN DIEGO, E. D. & TUCKER, W. G. (1967) Infusion of Fluorinated Pyrimidines into the Hepatic Artery for Treatment of Metastatic Cancer of the Liver. *Cancer, N.Y.*, **20**, 1886.

COSTANZI, J. J. & COLTMAN, C. A. (1969) Combination Chemotherapy using Cyclophosphamide, Vincristine, Methotrexate and 5-Fluorouracil in Solid Tumours. *Cancer, N.Y.*, **23**, 589.

DILLARD, B. M. (1969) Experience with Twenty-six Hepatic Lobectomies and Extensive Hepatic Resections. *Surgery, Gynec. Obstet.*, **129**, 249.

DONEGAN, W. L., HARRIS, H. S. & SPRATT, J. S. (1969) Prolonged Continuous Hepatic Infusion. *Archs Surg.*, **99**, 149.

FLANAGAN, L. & FOSTER, J. H. (1967) Hepatic Resection for Metastatic Cancer. *Am. J. Surg.*, **113**, 551.

FRACCHIA, A. A., RANDALL, H. T. & FARROW, J. H. (1970) The Results of Adrenalectomy for Advanced Breast Cancer in 500 Consecutive Patients. In *Breast Cancer Early and Late.* Ed. staff of M. D. Anderson Hospital. Chicago: Year Book Medical Publishers.

HANHAM, I. W. F., NEWTON, K. A. & WESTBURY, G. (1971) Seventy-five Cases of Solid Tumours Treated by a Modified Quadruple Chemotherapy Regime. *Br. J. Cancer*, **25**, 462.

INGOLD, J. A., REED, G. B., KAPLAN, H. S. & BAGSHAW, M. A. (1965) Radiation Hepatitis. *Am. J. Roentg.*, **93**, 200.

JAFFE, B. M., DONEGAN, W. L., WATSON, F. & SPRATT, J. S. (1968) Factors Influencing Survival in Patients with Untreated Hepatic Metastases. *Surgery, Gynec. Obstet.*, **130**, 773.

KARNOFSKY, D. A. & BURCHENAL, J. H. (1948) In *Evaluation of Chemotherapeutic Agents.* Ed. McCleod. New York: Columbia University Press.

LABELLE, J. J., LUCAS, R. J., EISENSTEIN, B., REED, M. D., VAITKEVICIUS, V. K. & WILSON, G. S. (1968) Hepatic Artery Catheterisation for Chemotherapy. *Archs Surg.*, **96**, 683.

LAHIRI, S. R., BOILEAU, G. & HALL, T. C. (1971) Treatment of Metastatic Colorectal Carcinoma with 5-Fluorouracil by Mouth. *Cancer, N.Y.*, **28**, 902.

LAWRENCE, W. (1965) Regional Cancer Chemotherapy: an Evaluation. *Prog. clin. Cancer*, **1**, 341.

MANSFIELD, C. M., KRAMER, S., SOUTHARD, M. E. & MANDELL, G. (1969) Prognosis in Patients with Metastatic Liver Disease Diagnosed by Liver Scan. *Radiology*, **93**, 77.

MURRAY-LYON, I. M., DAWSON, J. L., BLENDIS, L. M., PARSONS, V. A., LAWS, J. W. & WILLIAMS, R. (1970) Treatment of Secondary Hepatic Tumours by Ligation of Hepatic Artery and Infusion of Cytotoxic Drugs. *Lancet*, ii, 172.

RAPOPORT, A. H. & BURLESON, R. L. (1970) Survival of Patients Treated with Systemic 5-Fluorouracil for Hepatic Metastases. *Surgery, Gynec. Obstet.*, **130**, 773.

SMITH, R. (1964) Hepatectomy. *Proc. R. Soc. Med.*, **57**, 547.

SULLIVAN, R. D., NORCROSS, J. W. & WATKINS, E. (1964) Chemotherapy of Metastatic Liver Cancer by Prolonged Hepatic Artery Infusion. *New Engl. J. Med.*, **270**, 321.

SULLIVAN, R. D. & ZUREK, W. Z. (1965) Chemotherapy of Liver Cancer by Protracted Ambulatory Infusion. *J. Am. Med. Ass.*, **194**, 481.

WATKINS, E., KHAZEI, A. M. & NAHRA, K. S. (1970) Surgical Basis for Arterial Infusion Chemotherapy of Disseminated Cancer of the Liver. *Surgery, Gynec. Obstet.*, **130**, 580.

WILSON, R. E., PIRO, A. J., ALIAPOULIS, M. A. & MOORE, F. D. (1971) Treatment of Metastatic Breast Cancer with a Combination of Adrenalectomy and 5-Fluorouracil. *Cancer, N.Y.*, **28**, 962.